# A Phase 1 Study of the Safety, Tolerability, and Pharmacokinetics of Single and Multiple Oral Doses of V-7404 in Healthy Adult Volunteers

**DOI:** 10.1128/AAC.01029-21

**Published:** 2021-09-17

**Authors:** Martin K. Kankam, Jennifer M. Burns, Marc S. Collett, Michael L. Corrado, Jeffrey R. Hincks

**Affiliations:** a Altasciences, Overland Park, Kansas, USA; b ViroDefense, Chevy Chase, Maryland, USA; c Independent Consultant/Advisor, Perkasie, Pennsylvania, USA

**Keywords:** 3C protease inhibitor, antiviral, capsid inhibitor, clinical development, enterovirus, phase 1 study, pocapavir, poliovirus, V-073, V-7404

## Abstract

V-7404, a direct-acting enterovirus (EV) 3C protease inhibitor, is being developed as a treatment option for serious EV infections, including infections in immunodeficient people excreting vaccine-derived polioviruses. V-7404 may be combined with pocapavir (V-073), a capsid inhibitor, to treat these infections. A phase 1 single ascending dose (SAD; *n* = 36) and multiple ascending dose (MAD; *n* = 40) study was conducted to assess the safety, tolerability, and pharmacokinetics (PK) of V-7404 in healthy adult volunteers following oral doses starting at 200 mg and escalating to 2,000 mg once daily (QD) and 2,000 mg twice daily (BID). Adverse events (AEs), vital signs, electrocardiographic findings, physical examinations, clinical laboratory values, and PK of blood samples were assessed. No notable differences in demographic and baseline characteristics were observed across the dose cohorts. A total of 35/36 participants (97.2%) completed the SAD study (1 withdrew in the placebo group), and 37/41 participants (90.2%) completed the MAD study (1 withdrew from the 2,000 mg QD and 3 withdrew from the 2,000 mg BID cohorts). No serious AEs or deaths were reported. Treatment-emergent AEs were mild or moderate in severity. Oral doses of V-7404 in all cohorts were readily absorbed and showed no significant accumulation. PK exposure increased in an approximately dose-proportional manner and appeared to be independent of time. Overall, V-7404 was well tolerated and exhibited an acceptable safety and PK profile, supporting further clinical investigation of V-7404 for the treatment of serious EV infections.

## INTRODUCTION

Enteroviruses (EVs) are important human pathogens and a major cause of human disease (1, 2). EVs are small, nonenveloped viruses with a single-stranded positive-sense RNA genome and include polioviruses (PVs), coxsackieviruses (CVs), echoviruses (ECVs), EV-A71, and EV-D68 (1, 3, 4). EVs are spread primarily through the fecal-oral route; however, some species (e.g., EV-D68) can also be spread through respiratory secretions (1).

Nonpolio EVs cause approximately 10 to 15 million infections and thousands of hospitalizations annually in the United States (1) (see also https://www.cdc.gov/non-polio-enterovirus/). While most people infected with EVs are asymptomatic or only experience mild illness, some experience serious complications, especially infants and those who are immunocompromised (https://www.cdc.gov/non-polio-enterovirus/). Neonates and young children with EV infections are at increased risk for severe diseases (4–6). In addition, PV infections in immunodeficient people have the potential to develop into chronic viral infections, which can further evolve into severe diseases (7, 8).

Currently under development by ViroDefense (Chevy Chase, MD), V-7404 is a direct-acting antiviral agent that irreversibly binds the EV 3C protease active site, making the agent a promising treatment for serious EV infections, including infections in immunodeficient people excreting vaccine-derived PVs. Other serious EV infections with life-threatening disease include neonatal sepsis and myocarditis, acute flaccid myelitis or paralysis, and meningoencephalitis. V-7404 may be combined with the capsid inhibitor pocapavir (V-073) to treat these infections (9, 10).

In cell culture tests, V-7404 was active against all 83 members of an EV panel comprising 47 PV strains and 36 nonpolio EV strains with half-maximal effective concentration (EC_50_) values ranging from 0.004 μM to 6.25 μM (mean, 0.379 μM; 198 ng/ml) (ViroDefense, data not shown). V-7404 demonstrated potent activity against EV-D68 isolates (range, 0.004 to 0.027 μM), with greater variability against EV-A71 isolates (range, 0.022 to 0.509 μM). Activity against the ECV-11 isolates evaluated ranged from 0.032 μM (11) to 0.352 μM. V-7404 was notably less active against CV-A16 (5.99 μM). In addition, *in vitro* drug combination experiments have demonstrated the synergistic activity of V-7404 in combination with antiviral capsid inhibitors, including pocapavir (12).

The nonclinical safety profile of V-7404 was defined by 14-day repeat-dose oral toxicology studies in rats and dogs, as well as in *in vitro* genotoxicity assays. In the 14-day repeat oral toxicology studies, V-7404 was well tolerated at all dose levels and produced no adverse effects. The no observed adverse effect levels (NOAELs) were the highest dosages evaluated, 1,500 mg/kg/day. The genotoxicity study results for V-7404 were negative.

Prior human experience with V-7404 involved a single ascending dose (SAD) study in healthy adult males (*n* = 14) conducted by Agouron/Pfizer (a previous sponsor who sought to develop V-7404 as an antirhinovirus agent), which showed that oral doses ranging from 200 mg to 2,000 mg were well tolerated with no serious adverse events (SAEs) (11). The area under the plasma drug concentration-time curve (AUC) extrapolated to infinite time (AUC_0–inf_) and maximum plasma concentration (*C*_max_) parameters increased in approximate proportion to increasing dose. The V-7404 terminal elimination half-life (*t*_1/2_) values were 8.85 ± 6.15 h and 7.06 ± 3.27 h after single-dose administration of 1,000 mg and 2,000 mg, respectively. All adverse events (AEs) were mild. There were no participant withdrawals and no deaths. Furthermore, there were no clinically significant changes in vital signs, electrocardiogram (ECG) findings, and clinical laboratory values.

A phase 1, double-blind, randomized, placebo-controlled, SAD and multiple ascending dose (MAD) study was conducted to assess the safety, tolerability, and pharmacokinetics (PK) of V-7404 in healthy adult volunteers following oral administration of doses starting at 200 mg and escalating to doses of 2,000 mg once daily (QD) and 2,000 mg twice daily (BID) and in the presence or absence of food (food effect study).

## RESULTS

**FIG 1:**
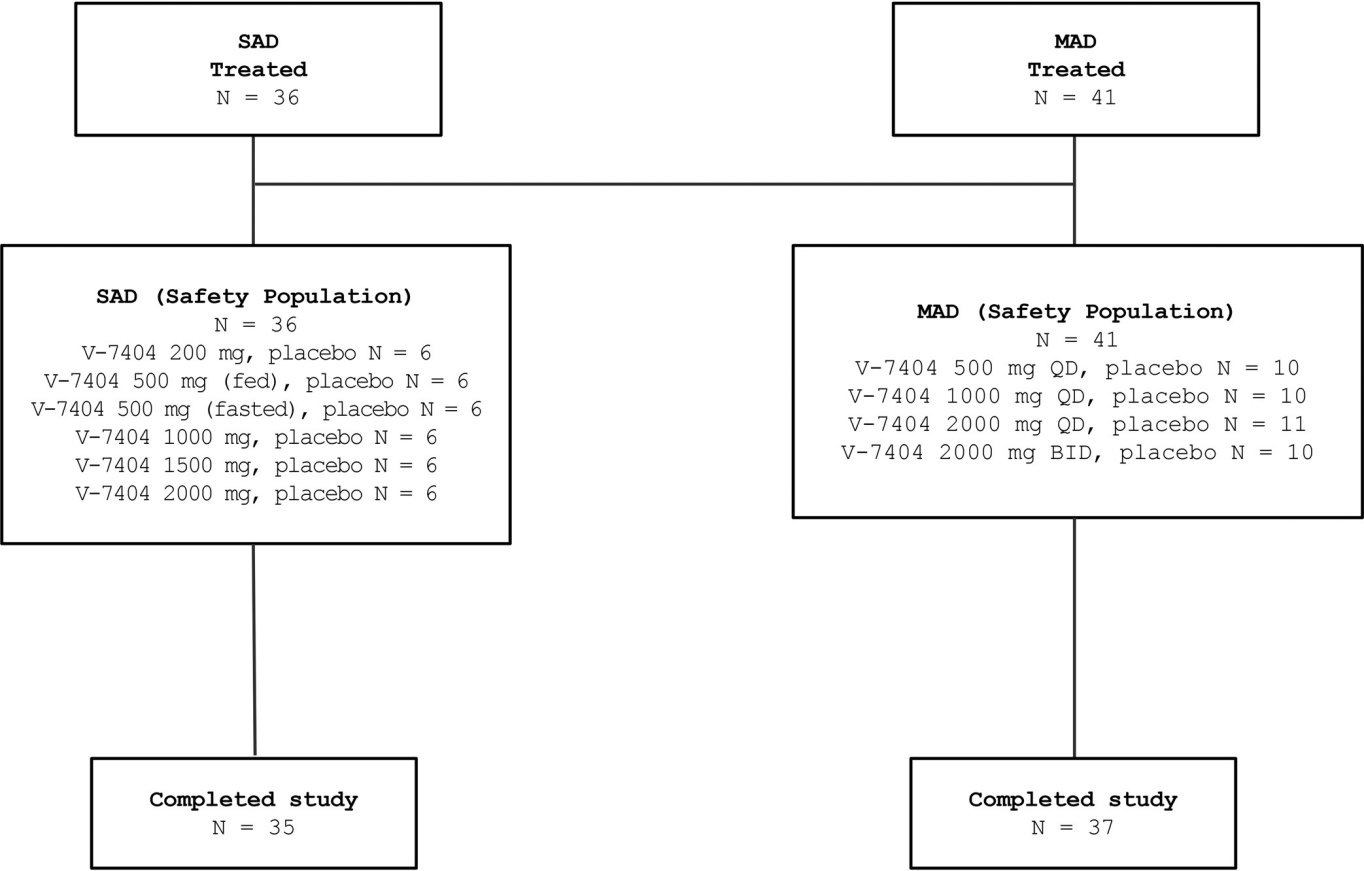
Disposition of study participants. SAD, single ascending dose; MAD, multiple ascending doses; QD, once daily; BID, twice daily.

Overall, 77 participants were enrolled, and 72 participants (93.5%) completed the phase 1 study (Fig. 1). In the SAD study, 36 participants were enrolled, and 35 participants (97.2%) completed the study. One participant in the placebo group withdrew and was discontinued on day 13 of the study. In the MAD study, 41 participants were enrolled, and 37 participants (90.2%) completed the study. Four participants were withdrawn and did not complete the study (1 participant in the 2,000 mg QD and 3 participants in the 2,000 mg BID V-7404 cohorts). One participant was withdrawn due to an AE at the investigator’s discretion, and study treatment was discontinued on day 10; one participant was withdrawn at the investigator’s discretion and study treatment was discontinued on day 11 as a result of coronavirus infection (coronavirus disease 2019 [COVID-19]) precautions (participant had a cough); one participant did not consume the entire study treatment and was discontinued on day 2; and one participant was withdrawn as a result of childcare issues related to COVID-19, and study treatment was discontinued on day 8.

No notable differences in participant demographic and baseline characteristics were observed across the different SAD and MAD cohorts (Tables 1 and 2).

**TABLE 1:** Demographics and baseline characteristics for single ascending dose safety population

Variable or category	Statistic^*a*^	No. (%) of patients							
		On placebo (*n* = 12)	On V-7404 at:						All participants (*n* = 36)
			200 mg (fasted, *n* = 4)	500 mg (fasted, *n* = 4)	500 mg (fed, *n* = 4)	1,000 mg (fasted, *n* = 4)	1,500 mg (fasted, *n* = 4)	2,000 mg (fasted, *n* = 4)	
Gender									
Male	*n* (%)	6 (50.0)	1 (25.0)	1 (25.0)	2 (50.0)	2 (50.0)	0 (0.0)	2 (50.0)	14 (38.9)
Female	*n* (%)	6 (50.0)	3 (75.0)	3 (75.0)	2 (50.0)	2 (50.0)	4 (100.0)	2 (50.0)	22 (61.1)
Race									
American Indian or Alaska Native	*n* (%)	0 (0.0)	0 (0.0)	0 (0.0)	0 (0.0)	0 (0.0)	0 (0.0)	0 (0.0)	0 (0.0)
Asian	*n* (%)	0 (0.0)	0 (0.0)	0 (0.0)	0 (0.0)	0 (0.0)	0 (0.0)	0 (0.0)	0 (0.0)
Black or African American	*n* (%)	5 (41.7)	1 (25.0)	3 (75.0)	0 (0.0)	1 (25.0)	3 (75.0)	2 (50.0)	15 (41.7)
Native Hawaiian or other Pacific Islanders	*n* (%)	0 (0.0)	0 (0.0)	0 (0.0)	0 (0.0)	0 (0.0)	0 (0.0)	0 (0.0)	0 (0.0)
White	*n* (%)	7 (58.3)	2 (50.0)	1 (25.0)	4 (100.0)	3 (75.0)	1 (25.0)	2 (50.0)	20 (55.6)
Multiple	*n* (%)	0 (0.0)	1 (25.0)	0 (0.0)	0 (0.0)	0 (0.0)	0 (0.0)	0 (0.0)	1 (2.8)
Other	*n* (%)	0 (0.0)	0 (0.0)	0 (0.0)	0 (0.0)	0 (0.0)	0 (0.0)	0 (0.0)	0 (0.0)
Ethnicity									
Hispanic or Latino	*n* (%)	1 (8.3)	1 (25.0)	0 (0.0)	1 (25.0)	0 (0.0)	0 (0.0)	0 (0.0)	3 (8.3)
Not Hispanic or Latino	*n* (%)	11 (91.7)	3 (75.0)	4 (100.0)	3 (75.0)	4 (100.0)	4 (100.0)	4 (100.0)	33 (91.7)
Age (yrs)	*n*	12	4	4	4	4	4	4	36
	Mean	31.3	35.3	31.3	23.3	30.0	26.8	32.0	30.3
	SD	7.3	5.6	3.4	4.3	5.5	3.8	10.1	6.7
	CV%	23.4	15.8	10.9	18.7	18.3	14.1	31.7	22.2
	Minimum	21	30	27	19	23	23	23	19
	Median	31	34	32	23	31	26	30	30
	Maximum	45	43	34	29	36	32	45	45
Ht (cm)	*n*	12	4	4	4	4	4	4	36
	Mean	168.71	167.00	170.63	171.63	164.25	162.43	167.75	167.76
	SD	7.89	6.23	2.36	13.59	4.99	4.19	13.23	8.09
	CV%	4.7	3.7	1.4	7.9	3.0	2.6	7.9	4.8
	Minimum	154.0	160.5	168.5	156.5	159.0	158.0	149.0	149.0
	Median	167.5	167.0	170.0	172.5	164.5	162.1	172.0	168.0
	Maximum	180.0	173.5	174.0	185.0	169.0	167.5	178.0	185.0
Wt (kg)	*n*	12	4	4	4	4	4	4	36
	Mean	73.70	82.93	67.55	65.93	71.80	67.15	76.45	72.54
	SD	13.97	13.95	10.45	9.30	13.36	14.71	15.88	13.27
	CV%	19.0	16.8	15.5	14.1	18.6	21.9	20.8	18.3
	Minimum	56.8	66.0	54.3	53.6	55.9	52.5	58.4	52.5
	Median	71.1	84.7	69.8	67.0	72.7	66.1	78.2	71.1
	Maximum	97.2	96.3	76.4	76.2	85.9	83.9	91.1	97.2
Body mass index (kg/m^2^)	*n*	12	4	4	4	4	4	4	36
	Mean	25.88	29.55	24.18	22.58	26.60	25.00	26.70	25.80
	SD	3.86	2.91	4.54	2.41	4.52	4.65	2.54	3.89
	CV%	14.9	9.9	18.8	10.7	17.0	18.6	9.5	15.1
	Minimum	20.0	25.7	19.3	19.8	22.3	20.1	23.7	19.3
	Median	25.4	30.3	23.8	22.5	26.8	24.6	26.8	25.7
	Maximum	31.7	32.0	29.9	25.6	30.5	30.7	29.5	32.0

a CV%, coefficient of variation.

**TABLE 2:** Demographics and baseline characteristics for the multiple ascending dose safety population

Variable or category	Statistic^*b*^	No. (%) of patients					
		On placebo (*n* = 8)	On V-7404 at^*a*^:				All participants (*N* = 41)
			500 mg QD, fasted (*n* = 8)	1,000 mg QD, fasted (*n* = 8)	2,000 mg QD, fasted (*n* = 9)	2,000 mg BID, fasted (*n* = 8)	
Gender							
Male	*n* (%)	3 (37.5)	4 (50.0)	4 (50.0)	4 (44.4)	4 (50.0)	19 (46.3)
Female	*n* (%)	5 (62.5)	4 (50.0)	4 (50.0)	5 (55.6)	4 (50.0)	22 (53.7)
Race							
American Indian or Alaska Native	*n* (%)	0 (0.0)	0 (0.0)	0 (0.0)	0 (0.0)	0 (0.0)	0 (0.0)
Asian	*n* (%)	0 (0.0)	0 (0.0)	0 (0.0)	1 (11.1)	0 (0.0)	1 (2.4)
Black or African American	*n* (%)	6 (75.0)	8 (100.0)	5 (62.5)	3 (33.3)	2 (25.0)	24 (58.5)
Native Hawaiian or other Pacific Islanders	*n* (%)	0 (0.0)	0 (0.0)	0 (0.0)	0 (0.0)	0 (0.0)	0 (0.0)
White	*n* (%)	2 (25.0)	0 (0.0)	3 (37.5)	5 (55.6)	6 (75.0)	16 (39.0)
Multiple	*n* (%)	0 (0.0)	0 (0.0)	0 (0.0)	0 (0.0)	0 (0.0)	0 (0.0)
Other	*n* (%)	0 (0.0)	0 (0.0)	0 (0.0)	0 (0.0)	0 (0.0)	0 (0.0)
Ethnicity							
Hispanic or Latino	*n* (%)	1 (12.5)	0 (0.0)	2 (25.0)	1 (11.1)	1 (12.5)	5 (12.2)
Not Hispanic or Latino	*n* (%)	7 (87.5)	8 (100.0)	6 (75.0)	8 (88.9)	7 (87.5)	36 (87.8)
Age (yrs)	*n*	8	8	8	9	8	41
	Mean	32.9	31.4	33.0	35.2	32.0	33.0
	SD	7.2	5.9	7.8	4.6	4.1	5.9
	CV%	22.0	18.7	23.5	13.0	12.9	17.9
	Minimum	25	19	20	27	27	19
	Median	33	32	33	37	33	33
	Maximum	44	38	43	41	40	44
Ht (cm)	*n*	8	8	8	9	8	41
	Mean	167.50	170.69	172.44	166.70	171.13	169.62
	SD	10.01	8.18	12.84	8.23	10.21	9.75
	CV%	6.0	4.8	7.4	4.9	6.0	5.7
	Minimum	155.0	156.0	158.0	148.0	152.0	148.0
	Median	166.0	173.8	168.3	167.0	173.8	168.5
	Maximum	186.0	178.0	194.0	178.0	183.0	194.0
Wt (kg)	*n*	8	8	8	9	8	41
	Mean	77.66	70.94	81.20	70.23	77.81	75.44
	SD	11.38	14.68	9.91	8.56	13.28	11.92
	CV%	14.7	20.7	12.2	12.2	17.1	15.8
	Minimum	60.8	56.0	71.7	57.7	49.2	49.2
	Median	76.4	68.4	78.0	68.4	82.5	75.3
	Maximum	98.1	94.7	100.9	86.5	93.8	100.9
Body mass index (kg/m^2^)	*n*	8	8	8	9	8	41
	Mean	27.40	24.61	27.53	25.44	26.46	26.27
	SD	2.09	5.01	2.40	3.45	3.12	3.40
	CV%	7.6	20.4	8.7	13.5	11.8	12.9
	Minimum	24.5	19.0	23.2	21.4	21.6	19.0
	Median	27.4	23.7	27.9	24.7	26.8	27.2
	Maximum	30.7	31.3	31.0	30.7	30.4	31.3

a BID, twice daily; QD, once daily.

b CV%, coefficient of variation.

All safety assessments were carried out using a safety population (Fig. 1). A total of 36 participants in the SAD study received a single administration of V-7404 or placebo at a dose of 200 mg, 500 mg (fasted), 500 mg (fed), 1,000 mg, 1,500 mg, or 2,000 mg, whereas a total of 41 participants in the MAD study received multiple administrations of V-7404 or placebo at a dose of 500 mg, 1,000 mg, or 2,000 mg QD or 2,000 mg BID.

In the SAD study, a total of 4/24 participants (16.7%) in the V-7404 group and 2/12 participants (16.7%) in the placebo group experienced mild treatment-emergent AEs (TEAEs). Only 1/12 participants (8.3%) in the placebo group and 1/24 participants (4.2%) in the V-7404 group experienced moderate TEAEs. Increased lipase levels (asymptomatic) were reported in one participant receiving 1,000 mg of V-7404. The lipase level had returned to normal the next time that it was assessed, 3 days later. There were no dose-related increases in TEAEs reported in the V-7404 group. No deaths or SAEs were reported.

In the MAD study, a total of 10/33 participants (30.3%) in the V-7404 group and 1/8 participants (12.5%) in the placebo group experienced mild TEAEs, and 2/33 participants (6.1%) in the V-7404 group and 0/8 participants (0.0%) in the placebo group experienced moderate TEAEs. In the V-7404 group, 2,000 mg BID cohort, 1/41 participants (2.4%) experienced increased lipase levels (asymptomatic), which led to discontinuation from the study treatment; this same participant also experienced increased amylase levels (asymptomatic). The amylase and lipase levels had returned to normal the next time that they were assessed, 2 days later. The majority of TEAEs were mild in severity, with the exception of three moderate TEAEs, namely increased lipase (asymptomatic) and increased amylase (asymptomatic) levels reported in 1 participant from the 2,000 mg BID cohort, and headache reported in 1 participant from the 2,000 mg QD cohort. The most prevalent TEAEs, occurring in 2 or 3 participants across the treatment groups, were mild in grade and included constipation, diarrhea, nausea, headache, and somnolence in the V-7404 group and headache in the placebo group. There were dose-related increases in the treatment-related TEAEs reported in the V-7404 group participants. As the dose increased, the proportion of related TEAEs increased from 12.5% (1,000 mg QD) to 22.2% (2,000 mg QD) and 75% (2,000 mg BID); however, no conclusions could be drawn because the sample size was small, and the study was not powered to evaluate this trend. The observed TEAEs will be monitored closely in subsequent clinical investigations. No deaths or SAEs were reported.

The PK assessment set consisted of 56/57 participants (98.2%) in the safety population who received V-7404. Of these 57 participants, 24/24 participants (100.0%) in the SAD study and 32/33 participants (97.0%) in the MAD study contributed PK data to the PK assessment set. One participant in the MAD study did not consume the entire first dose of V-7404, so the participant was withdrawn and did not contribute data to the PK assessment set. In the MAD study, 32 participants contributed PK data on day 1, and 29 participants contributed PK data on day 14.

**FIG 2:**
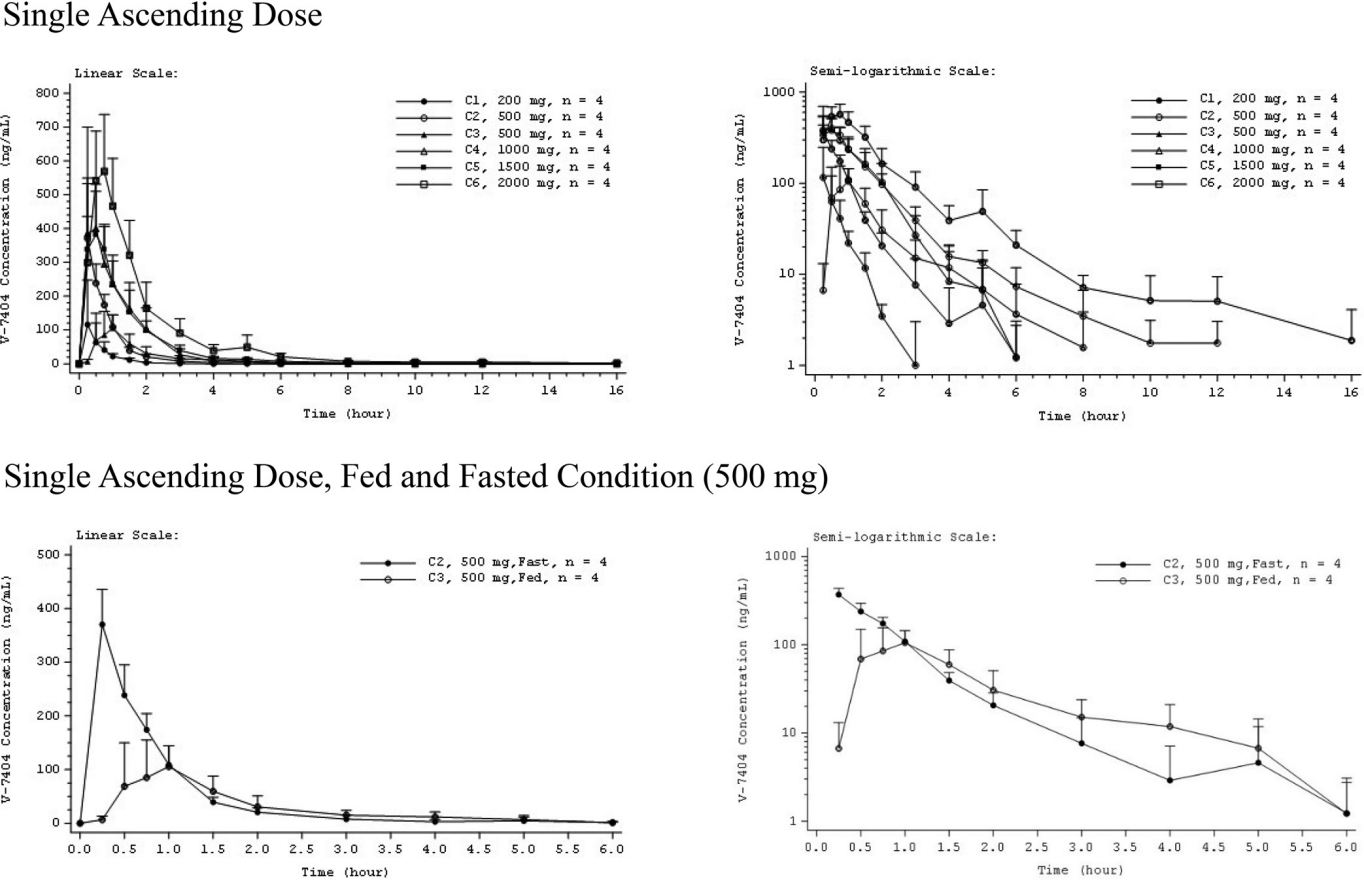
Mean V-7404 plasma concentration-time profiles for single ascending dose. Error bars indicate standard deviation (SD). Mean values BLQ were treated as zero for plotting purposes. The *x* axis is truncated at the time of the last quantifiable mean. BLQ, below the lower limit of quantification (LLOQ; 1.00 ng/ml); C, cohort.

PK profiles were similar across all the SAD and MAD cohorts. Mean plasma concentrations of SAD V-7404 peaked at 0.25 to 0.75 h across (fasted) cohorts and trended toward later times with increasing dose (Fig. 2). Individual V-7404 plasma concentrations declined in a monoexponential or biexponential manner following *C*_max_. The geometric mean V-7404 *t*_1/2_ and V-7404 apparent volume of distribution (*V_z_*/*F*) showed increasing trends with increasing dose level, which may have been due to concentrations falling below the lower limit of quantification (BLQ) earlier at the lower dose levels, resulting in incomplete estimation/representation of the terminal phase (Table 3). V-7404 time to maximum plasma concentration (*t*_max_) was similar across SAD cohorts, with median *t*_max_ of 0.25 to 0.63 h. Over a 10-fold increase in dose from 200 mg to 2,000 mg, geometric mean V-7404 AUC_0–inf_ and *C*_max_ increased by 14.2-fold and 7.6-fold, respectively.

**TABLE 3:** Descriptive statistics for V-7404 plasma pharmacokinetic parameters for single ascending dose and multiple ascending doses administered once daily (pharmacokinetic assessment set)

Cohort/dose (mg)	Statistic^*c*^	AUC (ng · h/ml)^*a*^	*C*_max_ (ng/ml)^*d*^	*t*_max_ (h)^*b*^	*t*_1/2_ (h)^*e*^	CL/*F* (liters/h)^*f*^	*V_z_*/*F* (liters)^*g*^	*C*_avg_ (ng/ml)^*h*^	LI^*i*^	RAUC^*j*^	RC_max_^*k*^
Single ascending dose, fasted condition				
1/200 (*n* = 4)	No. of patients	3	4	4	3	3	3				
	GeoMean	77.42	83.35	0.5	0.3152	2,580	1,170				
	GeoCV%	54.7	170.5	0.25, 1.50	4.1	54.8	50.7				
2/500 (*n* = 4)	No. of patients	4	4	4	4	4	4				
	GeoMean	277.1	366.6	0.25	0.8249	1,804	2,145				
	GeoCV%	22.8	16.6	0.25, 0.25	61.3	22.7	60.7				
4/1,000 (*n* = 4)	No. of patients	3	4	4	3	3	3				
	GeoMean	591.9	436.6	0.38	1.017	1,688	2,475				
	GeoCV%	53.8	55.2	0.25, 0.50	116	53.8	44.7				
5/1,500 (*n* = 4)	No. of patients	3	4	4	3	3	3				
	GeoMean	590.4	417	0.51	1.939	2,537	7,103				
	GeoCV%	19.8	18.4	0.25, 0.77	95.9	19.9	85.5				
6/2,000 (*n* = 4)	No. of patients	3	4	4	3	3	3				
	GeoMean	1,098	636.8	0.63	2.336	1,823	6,148				
	GeoCV%	21.2	18.8	0.25, 0.75	42.3	21.3	23.3				
Single ascending dose, fed and fasted conditions (500 mg)				
2/500 (*n* = 4), fasted	No. of patients	4	4	4	4	4	4				
	GeoMean	277.1	366.6	0.25	0.8249	1,804	2,145				
	GeoCV%	22.8	16.6	0.25, 0.25	61.3	22.7	60.7				
3/500 (*n* = 4), fed	No. of patients	3	4	4	3	3	3				
	GeoMean	139	124.7	1	0.6834	3,600	3,551				
	GeoCV%	43.9	39.9	0.50, 1.50	29.7	43.8	79.5				
Multiple ascending dose, day 1 QD				
1/500 (*n* = 8)	No. of patients	8	8	8	8	8	8				
	GeoMean	240.7	246.9	0.48	0.769	2,076	2,305				
	GeoCV%	49.8	60.6	0.25, 0.75	69	49.6	69.9				
2/1,000 (*n* = 8)	No. of patients	8	8	8	8	8	8				
	GeoMean	649.8	580.4	0.5	1.618	1,540	3,596				
	GeoCV%	35.3	37.7	0.25, 0.75	52.8	35.3	41.3				
3/2,000 (*n* = 8)	No. of patients	3	8	8	3	3	3				
	GeoMean	1075	938.9	0.5	3.845	1,860	10,320				
	GeoCV%	45.3	38.3	0.25, 1.00	71.3	45.4	23				
Multiple ascending dose, day 14 QD
1/500 (*n* = 8)	No. of patients	8	8	8	5	8	5	8	8	8	8
	GeoMean	302.4	370.2	0.25	1.414	1,656	3,022	12.58	1.254	1.258	1.499
	GeoCV%	32	36.3	0.25, 1.00	76	32.2	46.1	32.1	31	31	53.2
2/1,000 (*n* = 8)	No. of patients	8	8	8	7	8	7	8	8	8	8
	GeoMean	564.5	433	0.5	1.822	1,771	4,667	23.53	0.869	0.872	0.746
	GeoCV%	22.3	44.3	0.25, 0.53	104.5	22.3	95.5	22.4	20.1	20.1	42.6
3/2,000 (*n* = 8)	No. of patients	8	8	8	6	8	6	8	3	8	8
	GeoMean	961.6	789.7	0.5	4.186	2,081	11,460	40.02	0.7707	0.8168	0.8406
	GeoCV%	33.9	41.5	0.25, 0.75	58.6	33.9	61.7	33.9	23.9	19.5	33.7

a Area under the plasma drug concentration-time curve (AUC) values are extrapolated to an infinite time (AUC_0–inf_) for all groups except the multiple ascending dose, day 14 QD cohort, which is calculated over a dosing interval (AUC_0–tau_). For this cohort, CL/F is also given as apparent clearance at steady state (CL_ss_/*F*).

b Median, minimum, and maximum presented for time to maximum plasma concentration (*t*_max_).

c GeoCV%, geometric coefficient of variation; GeoMean, geometric mean; QD, once daily.

d *C*_max_, maximum concentration of drug in serum.

e *t*_1/2_, terminal elimination half-life.

f CL/*F*, apparent clearance.

g *V_z_*/*F*, apparent volume of distribution.

h *C*_avg_, average plasma concentration over a dosing interval.

i LI, linearity index.

j RAUC, accumulation ratio for AUC.

k RC_max_, accumulation ratio for *C*_max_.

Relative to food status, mean plasma concentrations of SAD V-7404 peaked later under the fed (at 1 h) than under the fasted (0.25 h) conditions (Fig. 2). Following *C*_max_, individual plasma concentrations declined in a monoexponential or biexponential manner under the fasted condition and in a monoexponential manner under the fed condition. Table 3 summarizes the plasma PK parameters for the fed and fasted conditions at the same dose level (500 mg). The V-7404 geometric mean AUC_0–inf_ was 50% lower for the fed (139.0 ng · h/ml) compared to the fasted (277.1 ng · h/ml) condition, and the V-7404 geometric mean *C*_max_ was 66% lower for the fed (124.7 ng/ml) compared to the fasted (366.6 ng/ml) condition.

**FIG 3:**
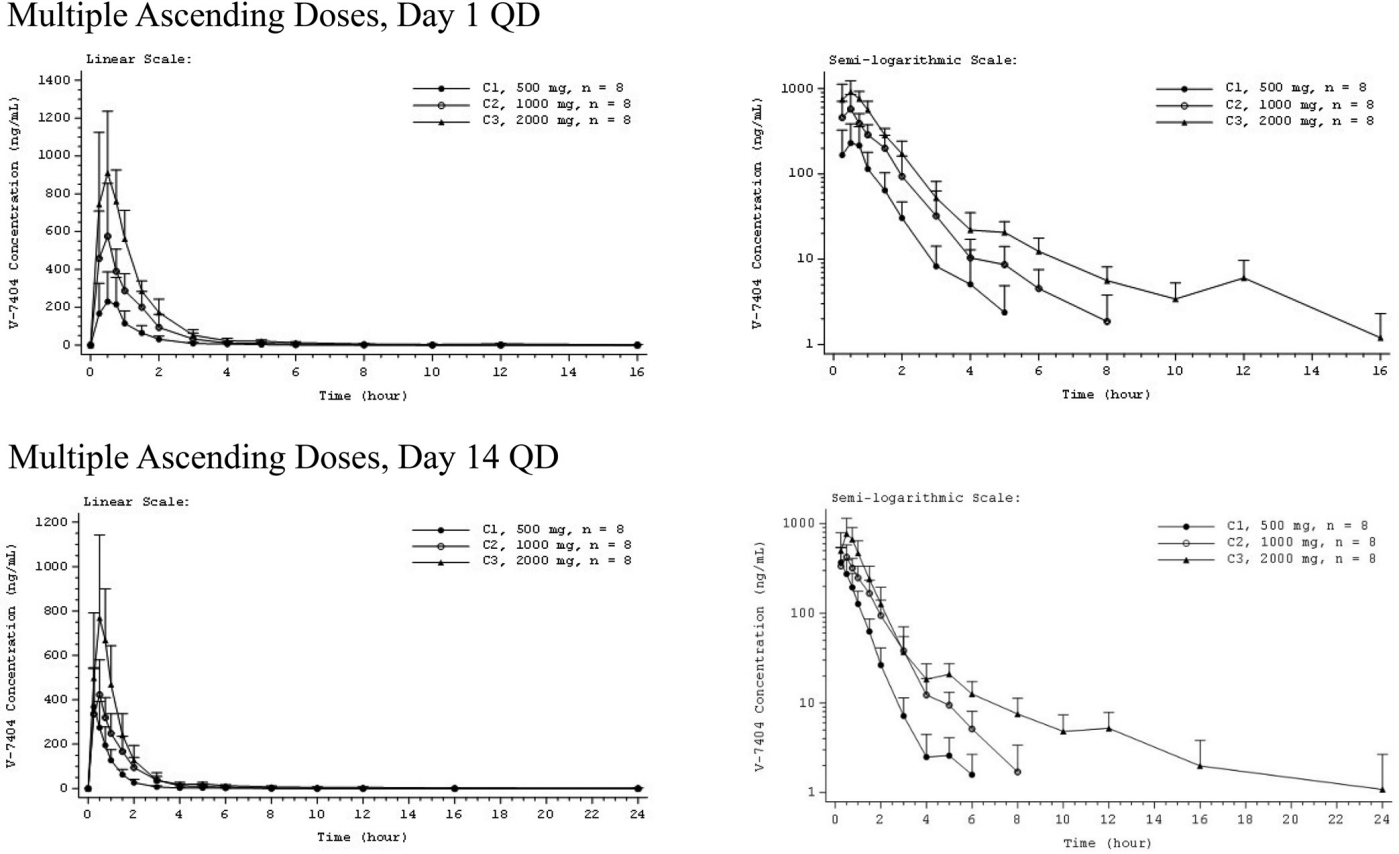
Mean V-7404 plasma concentration-time profiles for multiple ascending doses administered once daily (QD), day 1 and day 14. Error bars indicate SD. Mean values BLQ were treated as zero for plotting purposes. The *x* axis is truncated at the time of the last quantifiable mean. BLQ, below the lower limit of quantification (LLOQ; 1.00 ng/ml); C, cohort.

**FIG 4:**
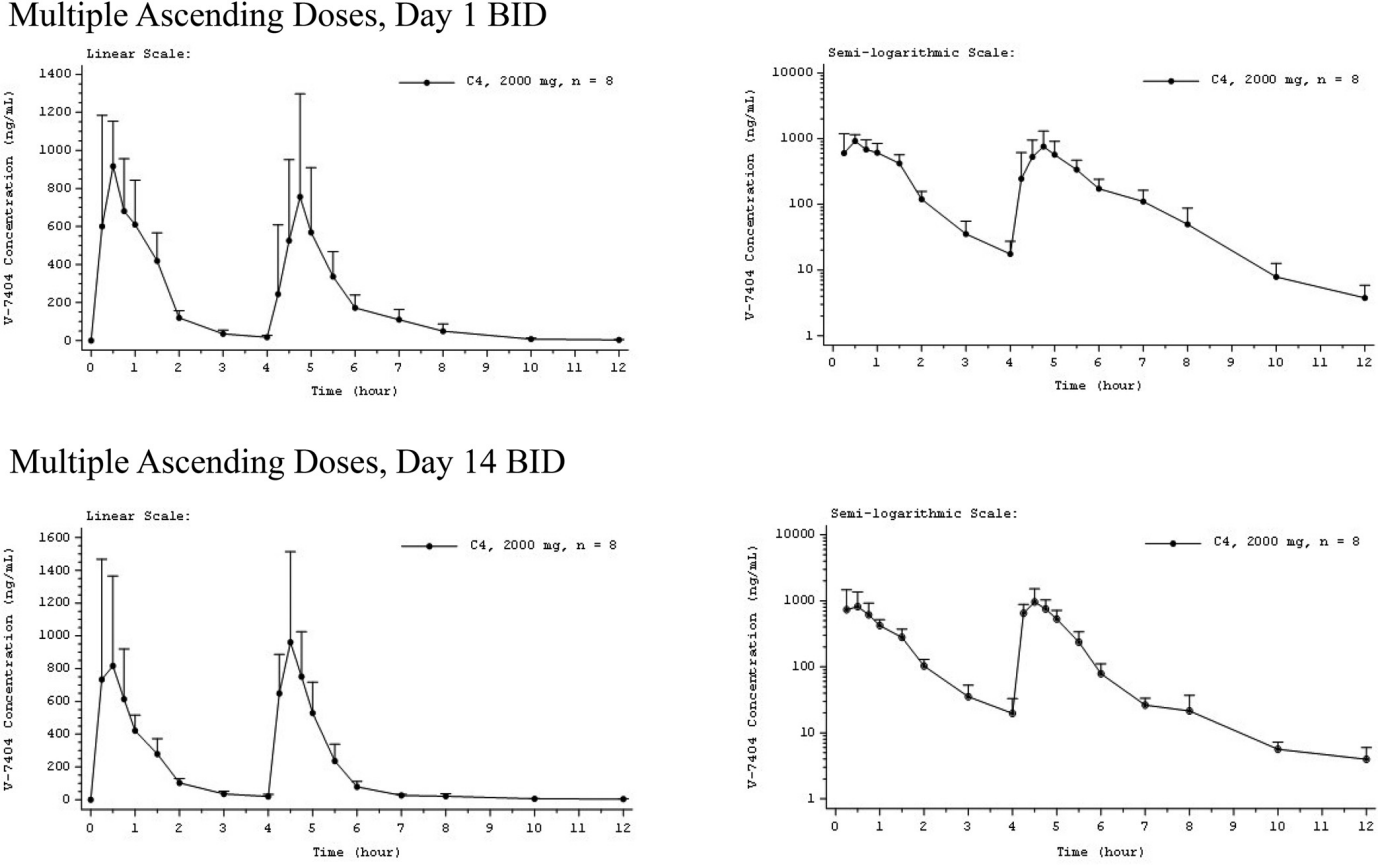
Mean V-7404 plasma concentration-time profiles for multiple ascending doses administered twice daily (BID), day 1 and day 14. Error bars indicate SD. Mean values BLQ were treated as zero for plotting purposes. The *x* axis is truncated at the time of the last quantifiable mean. Three participants were withdrawn before day 14 dosing, so *n* = 5 for the day 14 pharmacokinetic profile. BID, twice daily; BLQ, below the lower limit of quantification (LLOQ; 1.00 ng/ml); C, cohort.

For all the QD cohorts, mean plasma concentrations of MAD V-7404 peaked at 0.50 h on day 1 and at 0.25 to 0.50 h on day 14 (Fig. 3). Following *C*_max_, individual plasma concentrations on days 1 and14 declined in a monoexponential or biexponential manner. Likewise, following the first dose of the day, the mean plasma concentration of MAD V-7404 BID peaked at 0.50 h, and following the second dose of the day, the mean plasma concentration peaked at 0.75 h on day 1 and 0.50 h on day 14 (Fig. 4). These results were consistent with those for the QD regimen.

Mean and individual trough concentrations of MAD V-7404 QD measured on days 2, 3, 7, 11, and 14 were all BLQ for the 500 mg and 1,000 mg QD cohorts. For the 2,000 mg QD cohort, mean trough concentrations of V-7404 were either BLQ or were less than twice the lower limit of quantification (LLOQ) of 1.00 ng/ml, indicating negligible accumulation of V-7404 with multiple dosing. Table 3 summarizes the plasma PK parameters for MAD V-7404 QD, days 1 and 14. V-7404 *t*_1/2_ and *V_z_*/*F* on days 1 and 14 had increasing trends with increasing dose level. V-7404 *t*_max_ values were similar across dose levels and study days, with a median *t*_max_ of approximately 0.25 to 0.5 h. The V-7404 accumulation ratio for AUC (RAUC) and accumulation ratio for *C*_max_ (RC_max)_ on day 14 showed minimal (all ratios were less than 1.5) or no accumulation of V-7404 at all dose levels with QD dosing. In addition, the geometric mean linearity index (LI) for V-7404 QD was within approximately 0.77 to 1.25 for all cohorts, indicating time independence.

Mean trough concentrations of MAD V-7404 2000 mg BID measured on days 2, 3, 7, 11, and 14 were either BLQ or were less than twice the LLOQ of 1.00 ng/ml, indicating negligible accumulation with multiple dosing. Tables 4 and 5 summarizes the plasma PK parameters for the V-7404 2000 mg BID cohort, days 1 and 14. The V-7404 median *t*_max, BID_ on day 1 occurred during the first dosing interval of the day and on day 14 occurred during the second dosing interval of the day. Median *t*_max_ during the second dosing interval of the day [*t*_max(4–24 h), BID_] on days 1 and 14 was similar to median *t*_max_ during the first dosing interval of the day [*t*_max(0–4 h), BID_]. V-7404 geometric mean *C*_max_ during the first dosing interval of the day [*C*_max(0–4 h), BID_] on day 1 was similar to *C*_max_ on day 1 for the 2,000 mg QD regimen. The V-7404 geometric mean AUC_0–inf, BID_ and AUC over a dosing interval BID (AUC_0–tau, BID_) were consistent with approximately twice the corresponding parameters for the 2,000 mg QD regimen. The V-7404 geometric mean *t*_1/2, BID_ on days 1 and 14 was shorter than the *t*_1/2_ for the 2,000 mg QD regimen and for the SAD 2,000 mg cohort. Similarly to the QD regimen, V-7404 RAUC and RC_max_ on day 14 showed no accumulation with BID dosing.

**TABLE 4:** Descriptive statistics for V-7404 plasma pharmacokinetic parameters for multiple ascending doses administered twice daily, day 1 (pharmacokinetic assessment set)

Cohort/dose (mg)	Statistic	AUC_0–inf_ (ng · h/ml)	*C*_max_ (ng/ml)	*C*_max(0–4 h)_ (ng/ml)^*b*^	*C*_max(4–24 h)_ (ng/ml)^*b*^	*t*_max_ (h)^*a*^	*t*_max(0–4 h)_ (h)^*a*^	*t*_max(4–24 h)_ (h)^*a*^	*t*_1/2_ (h)	CL/*F* (liters/h)	*V_z_*/*F* (liters)
4/2,000 BID (*n* = 8)	*n*	7	8	8	8	8	8	8	7	7	7
	GeoMean	2011	1018	988.5	670.3	0.63	0.50	0.75	1.320	1,987	3,780
	GeoCV%	38.3	31.5	33.3	70.8	0.25, 4.75	0.25, 0.75	0.50, 1.50	69.2	38.4	50.2

a Median, minimum, and maximum presented for *t*_max_. *t*_max_, time to *C*_max_; *t*_max(0–4 h)_, *t*_max_ during the first dosing interval of the day; *t*_max(4–24 h)_, *t*_max_ during the second dosing interval of the day.

b *C*_max(0–4 h)_, *C*_max_ during the first dosing interval of the day; *C*_max(4–24 h)_, *C*_max_ during the second dosing interval of the day.

**Table 5:** Descriptive statistics for V-7404 plasma pharmacokinetic parameters for multiple ascending doses administered twice daily, day 14 (pharmacokinetic assessment set)

Cohort/dose (mg)	Statistic^*b*^	AUC_0–tau_ (ng · h/ml)	*C*_max_ (ng/ml)	*C*_max(0–4 h)_ (ng/ml)	*C*_max(4–24 h)_ (ng/ml)	*t*_max_ (h)^*a*^	*t*_max(0–4 h)_ (h)^*a*^	*t*_max(4–24 h)_ (h)^*a*^	*t*_1/2_ (h)	CL_ss_/*F* (liters/h)	*V_z_*/*F* (liters)	*C*_avg_ (ng/ml)	RAUC	RC_max_
4/2,000 BID (*n* = 8)	*n*	5	5	5	5	5	5	5	4	5	4	5	5	5
	GeoMean	1883	1011	827.7	896.7	4.25	0.25	0.50	2.270	2125	6542	78.55	0.9987	0.9799
	GeoCV%	35.4	41.0	56.5	55.8	0.25, 4.50	0.25, 0.75	0.25, 0.52	110.6	35.3	157.6	35.6	17.2	22.8

a Median, minimum, and maximum presented for *t*_max_. *t*_max_, time to *C*_max_; *t*_max(0–4 h)_, *t*_max_ during the first dosing interval of the day; *t*_max(4–24 h)_, *t*_max_ during the second dosing interval of the day.

b Three participants were withdrawn before day 14 dosing, so *n* = 5 for the day 14 pharmacokinetic profile.

## DISCUSSION

Overall, in the phase 1 SAD and MAD study, V-7404 dosing up to 2,000 mg for 14 days QD and BID was well tolerated and exhibited an acceptable safety and PK profile, supporting further clinical investigation of V-7404 for the treatment of serious EV infections.

Relative to the safety profile, TEAEs reported across the SAD and MAD studies were mild or moderate in severity. One participant in the MAD study experienced a TEAE, which led to discontinuation of the study treatment. There were 2 incidences of clinically significant abnormal blood chemistry parameters, namely, in the SAD study, one participant with increased lipase levels (asymptomatic) in the 1,000 mg cohort and, in the MAD study, one participant with increased lipase (asymptomatic) and amylase (asymptomatic) levels in the 2,000 mg BID cohort. The participant in the SAD study had elevated lipase 4.0× the upper limit of normal (ULN; range, 0.1167 to 1.0002 uKat/liter) on day 2, which returned to the normal range 3 days later when evaluated at a follow-up, unscheduled assessment. The participant in the MAD study entered the study with elevated amylase (1.1× ULN; range, 0.3507 to 1.6867 uKat/liter), which was considered by the clinical investigator to be not clinically significant, while prestudy lipase levels were within the normal range. Blood chemistry was assessed per protocol on days 3 and 7. On day 3, both amylase and lipase levels were within the normal range; however, on day 7, amylase was 2.7× ULN and lipase was 4.5× ULN. Two days later, a follow-up, unscheduled assessment showed that both amylase and lipase were within the normal range. No signs of pancreatic abnormalities were noted in preclinical evaluations of V-7404. Amylase and lipase will be monitored during the continued clinical development of V-7404.

Relative to the PK profile, V-7404 in single or multiple oral doses up to 2,000 mg daily for 14 days was well-tolerated in healthy adult volunteers. V-7404 administered orally was readily absorbed, with a median *t*_max_ of 0.25 to 0.63 h and a *t*_1/2_ of 0.32 to 2.34 h across single-dose fasted cohorts. Individual V-7404 plasma concentrations declined in a monoexponential or biexponential manner following *C*_max_; however, the profiles with monoexponential decline appeared to be due to censoring of the data caused by concentrations falling below the lower limit of quantification of the assay prior to the later phase. V-7404 *t*_1/2_ had increasing trends with dose level, but these trends may have been an artifact of this assay sensitivity issue, caused by characterizing a different (earlier) phase or a transitional area between phases in the profiles of the lower versus higher dose levels. Although variability and potential underestimation of exposure at the lower doses made interpretation of proportionality less clear in some cases, PK exposure for V-7404, particularly AUC, increased in an approximately dose-proportional manner over the dose ranges studied. Furthermore, following multiple doses QD and BID, V-7404 showed little or no accumulation, and the PK of V-7404 appeared to be independent of time for QD and BID dosing. Finally, V-7404 plasma geometric mean AUC_0−inf_ and *C*_max_ were 50% and 66% lower, respectively, for the fed compared to the fasted condition, and median V-7404 *t*_max_ was later for the fed (1.00 h) compared to the fasted (0.25 h) condition.

## MATERIALS AND METHODS

The study protocol and all other appropriate study-related information were reviewed and approved by Midlands Independent Review Board (Overland Park, KS). The study was conducted in accordance with The Declaration of Helsinki, International Council for Harmonisation Good Clinical Practice Guidelines, and country-specific laws and regulations. Written informed consent was obtained from all study participants during screening and prior to the initiation of any study-related procedures.

The phase 1 study was conducted in two parts at a single site in the United States from December 2018 to April 2020: approximately 36 healthy adult volunteers were planned to participate in a double-blind, randomized, placebo-controlled, SAD study with oral V-7404, and approximately 40 healthy adult volunteers were planned to participate in a double-blind, randomized, placebo-controlled, MAD study with oral V-7404. Participants were randomized in a 2:1 ratio to receive SAD V-7404 or matching placebo. Six cohorts (*n* = 36) each comprised 2 participants randomized to placebo and 4 participants randomized to V-7404 at 200 mg, 500 mg (fasted and fed), 1,000 mg, 1,500 mg, and 2,000 mg. Participants were randomized in a 4:1 ratio to receive MAD V-7404 or matching placebo QD or BID for 14 days. Four cohorts (*n* = 40) each comprised 2 participants randomized to placebo and 8 participants randomized to V-7404 at 500 mg, 1,000 mg, and 2,000 mg QD and 2,000 mg BID (administered 4 h apart) cohorts. The 4-h interval was specified in the protocol, as dose and regimen are being evaluated for treatment in potential combination with another antiviral agent, pocapavir. Table 6 summarizes the planned doses and cohorts for the SAD and MAD study. V-7404 or placebo was supplied as granules and was administered orally as a suspension in water.

**TABLE 6:** Study design and planned doses

Dose group	Cohort	Dose level (mg)^*b*^	No. of healthy adult volunteers given the drug or placebo^*a*^		Prandial state
			V-7404	Placebo	
Single ascending dose (SAD)	1	200	4	2	Fasted
	2	500	4	2	Fasted
	3	500	4	2	Fed
	4	1,000	4	2	Fasted
	5	1,500	4	2	Fasted
	6	2,000	4	2	Fasted
Multiple ascending doses (MAD)	1	500 QD	8	2	Fasted
	2	1,000 QD	8	2	Fasted
	3	2,000 QD	8	2	Fasted
	4	2,000 BID	8	2	Fasted

a All groups contained males and females.

b QD, once daily; BID, twice daily (administered 4 h apart).

Allocation to treatment was predetermined using a randomization code. A separate randomization list was generated for the SAD and MAD study, so that each part could be unblinded independently if necessary. Except for the pharmacists, the statistician preparing the randomization, the biological analysts, and the quality assurance auditors where necessary, all clinical and nonclinical staff, including the data management staff, were blind to treatment allocation until after the database was locked for the phase 1 study.

### Study objectives.

The primary objectives of the SAD study were to assess the safety and tolerability of single oral doses of V-7404 administered to healthy adult volunteers and to assess the plasma PK of V-7404 after administration of single oral doses. The secondary objective of the SAD study was to assess the effects of food on the safety, tolerability, and single-dose PK of V-7404. The primary objectives of the MAD study were to assess the safety and tolerability of multiple oral doses of V-7404 administered to healthy adult volunteers, both QD or BID, for 14 days, and to assess the PK of V-7404 after administration of single and multiple oral doses.

### Dose selection.

Initially, for the phase 1 SAD and MAD study of V-7404, participants were administered single oral doses starting at 200 mg and escalating to 2,000 mg. Safety exposure levels from the study of dogs (i.e., NOAELs from the toxicity study) and of the human highest dose from the previous SAD study (11) were calculated based on estimated exposures that could be achieved in this SAD and MAD study.

### Participant selection.

Healthy adults of any ethnicity, race, or gender, aged between 18 to 45 years (inclusive) and with a body mass index between 18.5 and 32 kg/m^2^ (inclusive) were eligible to participate in the study. In addition, if of childbearing potential, participants were agreeable to using one of the accepted contraceptive regimens for the durations specified. Participants were healthy as determined by medical evaluation, including medical history, physical examination, laboratory tests, and ECG, with no evidence of any disease or condition that might have compromised the cardiovascular, hematological, renal, hepatic, pulmonary, endocrine, central nervous, or gastrointestinal systems.

Participants were excluded if they had a clinically significant abnormal ECG; clinically significant laboratory values (e.g., aspartate aminotransaminase [AST]/serum glutamic oxaloacetic transaminase [SGOT] > 20% above the ULN); a history of alcoholism or drug addiction within 2 years of the study start; a history of serious mental illness; a history of difficulty donating blood or inadequate venous access; a history of smoking within <6 months of screening; prescription or over-the-counter (OTC) drug use within 14 days of the first dose of the study treatment (or for drugs with a *t*_1/2_ greater than 10 days, within 60 days); or positivity for human immunodeficiency virus type 1 and type 2 (HIV-1/HIV-2) antibodies, hepatitis B surface antigen (HBsAg), or hepatitis C antibody.

### Study procedures.

In the SAD study, there were up to 5 dose levels with 6 participants per level (4 V-7404 and 2 placebo), except for the 500-mg dose level, which included two separate cohorts of 6 participants each for assessing fasted and fed states (Table 6). Participants were required to visit the clinic at screening and end of study (EOS; day 14), and were confined to the clinic from days −1 to 2. Dosing began at the lowest level and proceeded sequentially to the next higher dose upon satisfactory review of safety, tolerability, and PK. The initial planned dose levels were 200 mg and 500 mg in the fasted state with one additional group of 6 participants (4 V-7404 and 2 placebo) dosed at a level of 500 mg in the fed state, so that the effect of food on the disposition of V-7404 could be evaluated.

Blood samples were obtained at prespecified time points over a 36-h period to analyze the PK of V-7404. Based on the interim analysis of PK in participants at a dose level of 500 mg in the fasted state that had an increased exposure and less variability than those given 500 mg in the fed state, the decision was made that subsequent dose levels were to be evaluated in the fasted state. Based on a review of the safety, tolerability, and PK profiles for the first three cohorts, 4, 5, and 6 were planned to receive 1,000 mg, 1,500 mg, and 2,000 mg, respectively, in the fasted state. Upon completion of the SAD study, the data were compiled and analyzed, and the results were reviewed before proceeding with the MAD study.

In the MAD study, there were up to 4 dose levels with 10 participants per level (8 V-7404 and 2 placebo) (Table 6). The dose levels and frequency were determined from the SAD study. Participants were required to visit the clinic at screening and EOS (day 28), and were confined to the clinic from days −1 to 15. V-7404 or placebo was administered orally QD or BID for 14 days. Blood samples were obtained at prespecified time points over a 24-h period on day 1 and on day 14 to analyze the PK of V-7404. Predose blood samples were obtained on days 3, 7, and 11 to determine V-7404 trough concentrations. Based on the safety, tolerability, and exposure achieved in the SAD study, the proposed dose levels for the MAD study were 500 mg, 1,000 mg, and 2,000 mg QD in the fasted state, as well as 2,000 mg BID (administered 4 h apart) in the fasted state.

Safety and tolerability assessments included monitoring AEs, measuring vital signs, recording ECGs, assessing clinical laboratory values from blood samples, and performing physical examinations. AEs were collected throughout the SAD and MAD studies, i.e., from predose until EOS, coded using Medical Dictionary for Regulatory Activities, version 21.1, and graded based on National Cancer Institute Common Terminology Criteria for Adverse Events, version 5.0 (or higher) (13).

The PK assessments included collecting blood samples and determining V-7404 concentrations in plasma. PK parameters were derived from these plasma concentrations. Plasma samples were analyzed for V-7404 using a validated liquid chromatography with tandem mass spectrometry (LC-MS/MS) analytical method based on turbo ion spray LC-MS. The LLOQ for V-7404 was 1.00 ng/ml using a 50-μl aliquot of human plasma and the upper limit of quantification was 1,000 ng/ml. The internal standard V-7404-d_5_ was used for the analyte.

### Study end points.

The primary safety end points included incidence of AEs, changes in laboratory results, ECGs, and vital signs in both the SAD and MAD study. The primary SAD PK end points were the plasma PK of V-7404, which included *C*_max_, *t*_max_, AUC from zero (dosing) to time of last quantifiable concentration (AUC_0–last_), dose-normalized AUC_0–last_ (AUC_0–last_/*D*), AUC_0–inf_, dose-normalized AUC_0–inf_ (AUC_0–inf_/*D*), AUC from zero (dosing) to 24 h (AUC_0–24 h_), dose-normalized AUC_0–24 h_ (AUC_0–24 h_/*D*), terminal elimination rate constant (λ_z_), *t*_1/2_, *V_z_*/*F*, and apparent clearance (CL/F). The secondary SAD PK endpoint was the dose proportionality of V-7404. Geometric means were considered the main measurements for PK parameters. AUC and *C*_max_ and the ratio of geometric means with confidence intervals were generated to assess gender and food effect, respectively.

The primary MAD PK end points were the single and multiple dose plasma PK of V-7404. MAD PK parameters for day 1 included *C*max, *t*_max_, AUC_0–tau_, and dose-normalized AUC_0–tau_ (AUC_0–tau_/*D*). MAD PK parameters on day 14 included *C*_max_, *t*_max_, minimum plasma concentration (*C*_min_), average plasma concentration over a dosing interval (*C*_avg_), fluctuation index (FI), AUC_0–tau_, AUC_0–last_, λ_z_, *t*_1/2_, *V_z_*/*F*, apparent clearance at steady state (CL_ss_/*F*), LI, RAUC, and RC_max_. The secondary MAD PK endpoint was the dose proportionality of V-7404 on day 14.

### Statistical methods.

Sample size or power for the SAD or MAD study cohorts was not formally calculated. A sample size of 6 participants (4 V-7404 and 2 placebo) per cohort in the SAD study and 10 participants (8 V-7404 and 2 placebo) per cohort in the MAD study was a feasible sample size to assess safety and PK. Two study populations were analyzed in the phase 1 study, the safety population, which comprised all participants who received at least 1 dose of V-7404 or placebo, and the PK assessment set, which included all participants who received V-7404 and had at least one quantifiable concentration at a scheduled PK time point after the start of dosing for at least one PK analyte. Safety and PK data were presented in tabular and/or graphical format and summarized using descriptive statistics. Dose proportionality was assessed using PK summary tables and figures without inferential analysis. The tables, graphs, and summaries were generated using SAS, version 9.4 (SAS Institute, Cary, NC). Noncompartmental PK parameter calculations were performed using Phoenix WinNonlin, version 8.0 (Certara, Princeton, NJ), or SAS.
